# Perioperative Duloxetine and Etoricoxibto improve postoperative pain after lumbar Laminectomy: a randomized, double-blind, controlled study

**DOI:** 10.1186/s12871-017-0450-z

**Published:** 2017-12-02

**Authors:** Josef Zekry Attia, Haidy Salah Mansour

**Affiliations:** Departments of Anesthesiology and I.C.U Al-Minia University, Faculty of Medicine, Minia University, Minia, 61111 Egypt

**Keywords:** Postoperative pain, Analgesia, Duloxetine, Etoricoxib, Lumbar Laminectomy

## Abstract

**Background:**

Duloxetine, Etoricoxib and opioid are of the commonly administered drugs in Lumbar laminectomy. The aim of this study is to assess the effect of perioperative use of Duloxetine in combination with Etoricoxib on postoperative pain and opioid requirements.

**Methods:**

One hundred twenty patients with ASA physical status were enrolled with age between 18 and 70 years. Patients were divided randomly into four groups of 30 patients: group P received placebo, group E received etoricoxib 120 mg, group D received duloxetine 60 mg and group D/E received duloxetine 60 mg capsules and etoricoxib 120 mg; 1 h before surgery and 24 h after.

**Results:**

Neither Duloxetine nor etoricoxib individually had effect on pain with movement, while their combination revealed a significant reduction in pain scores over the entire postoperative period at rest and on movement. Etoricoxib showed a significant decrease in pain at all times at rest when compared with group P, while it showed significant pain decrease only at 0, 2 and 4 h when compared with group D. On the other hand duloxetine alone showed significant decrease in pain at rest at 24 h and 48 h when compared with group P. ConcerningMorphine requirement after 24 h.; it wassignificantly lower in the D/E group in comparison with groups P, E and D. It should be noted also that there was a significant decrease morphine requirement in both groups E and D.

**Conclusion:**

The perioperative administration of the combination of etoricoxib and duloxetine improved analgesia and reduced opioid consumption without significant side effects.

**Trial registration:**

ISRCTN48329522. 17 June 2017

## Background

Postoperative pain is mediated by different mechanisms at multiple neural sites. Thus, multimodal analgesics can reduce the postoperative pain [1]. Although Opioids are considered the analgesics of choice to treat moderate to severe pain, their use carries the risk of side effects and hyperalgesia [2]. Multimodal analgesia can be achieved by combining different analgesics and different methods of administration, to provide better analgesia synergistically compared with conventional analgesia [3]. Therefore,lower doses for each drug can be provided with fewer overall side-effects obtained from individual compounds [4].

Recently, antidepressants such as duloxetine, a selective serotonin and norepinephrine reuptake inhibitor (SSNRI), have accomplished pain relief in persistent and chronic pain as in fibromyalgia, postherpetic neuralgia, diabetic neuropathy [5], osteoarthritis and musculoskeletal pain [6]. Theanalgesic effect of duloxetine is attributed to its ability to enhance both serotonin and norepinephrine neurotransmission in descending inhibitory pain pathways. [7]. Moreover, some studies have promoted its use to improve the quality of recovery after surgery and reduce the acute postoperative pain after knee replacement surgery [8], mastectomy [9], hysterectomy [10], and after spine surgery [11]. In addition it can improve postoperative quality of recovery through mood improvement that can be helpful in the postoperative period [12].

Another group of analgesics isthe non-steroidal anti-inflammatory drugs (NSAIDs) which are used for acute pain management. It has pain-relieving, antipyretic, and anti-inflammatory properties [13]. It’s thought that its analgesic effect is caused by suppression of cyclooxygenase (COX) thus it inhibits the synthesis of PGs [14]. However, beingnonselective in inhibition of COX1 and COX2; several adverse effects can appear [15]. It is thought that the therapeutic activity of NSAIDs is due to the inhibition of COX-2, whereas the adverse effects results from inhibition of COX-1 [16]. Thus, many studies show that the selective COX-2 inhibitors have a great role in reducing the postoperative pain and reducing the dose of postoperative opioid consumption [17–19].

Etoricoxib is more highly selective of COX-2 over COX-1 than celecoxib [20], and characterized bylonger duration of action ranging 22–24 h. In addition, it is absorbed rapidly after oral intake so the peak plasma concentrations are reached after 1 h [21]. It was examined preoperatively by different studies and revealedefficacy in providing postoperative analgesia after abdominal [17], laparoscopic [19], gynecological [22] and orthopedic procedures [18, 23]. However, additive or synergistic interactions can be detected when two analgesics are administered together at the same time [24]. In cases of synergistic interaction, we can use smaller doses of each drug to achieve good analgesia with fewer adverse effects derived from individual compounds [4].

The main objective of the present study was to examine perioperativelythe analgesic efficacy with the combination of duloxetine and etoricoxib on postoperative pain and itsopioid-sparing properties when given as part of a multimodal pain strategy in patients undergoing surgery on the lumbar spine. In addition toevaluating the patient’s satisfaction and the adverse effects related to the combination of both medications.

## Methods

After institutional Ethics Committee approval, this prospective double-blind, randomized, controlled study was started in November 2015 at the department of anesthesia and intensive care unit; El- Minia University Hospital. The study involved 120 adult patients of both genders aging between 18 and 70 years of age with an ASA physical status of I, II and III,who were scheduled for single level lumbar spinal disc prolapse surgery. All patients gave written informed consent.

Exclusion criteria involved patients with history of allergic reaction to any of the study drugs, history of drug or alcohol abuse, and abnormal renal or liver function tests. Patients using antidepressants had to stop taking them 2 weeks before surgery. Also, Patients with previous cervical surgeries, psychiatric disorders and patients receiving opioid analgesic medications within 24 h preoperatively were excluded.

We asked the patients to visit the outpatient clinic 1 day before surgery for assessment and performing laboratory investigations. We also explained to them the study protocols, including analgesic administration and the11-point numeric rating scale (NRS) where 0 being ‘no pain’ and ‘10’ being the maximal worst pain [25].

### Study design

The patients admitted to the hospital were randomized according to the computer-generated random numbers with closed-sealed envelopes into one of the four groups 30 patients each. The study medications were prepared by the pharmacy of the hospital and given to the patients by an investigator not involved in the study. They were duloxetine 60 mg capsules (Cymbalta; Eli Lilly & Company, Indiana, USA), etoricoxib 60 mg film coated tablets (Arcoxia; Merck Sharp &Dohme Limited, Hertford road, Hoddesdon, Hertfordshire, UK), and placebo capsules that matched the duloxetine capsules or etoricoxib tablet in color and size. All drugs were given 1 h before surgery and repeated after 24 h.

1. The Group P (Placebo) received placebo capsule + two placebo tablet
2. The Group E (etoricoxib) received placebo capsule + two etoricoxib tablet 60 mg
3. The Group D (duloxetine) received duloxetine capsule 60 mg + two placebo tablet
4. The Group DC (duloxetine + etoricoxib) received duloxetine 60 mg capsules + two etoricoxib tablets 90 mg

On arrival to the operating room,standard intraoperative monitoring includedelectrocardiogram (ECG), heart rate (HR), mean arterial blood pressure (MABP), oxygen saturation (SPO2) and end tidal CO2 were recorded and subsequent measurements were recorded every 5 min till the end of the operation using a multiparameter monitor (Mindray iMEC12, Hi-tech industrial Park,Nanshan, Shenzhen, china).

General anesthesia was induced by (1.5 μg/kg) fentanyl IV, (2 mg/kg)propofol IV, and endotracheal intubation was performed with (0.5 mg/kg) IV atracurium. Maintenance of anesthesia was done through inhalation of a mixture of oxygen (3 L/min) (1–2%) isoflurane and (0.05 mg/kg) atracurium as intermittent dose of muscle relaxant to ensure proper muscle relaxation during the procedure.

An anesthetist who was blinded to the groups took all the measurements. Their goal was to adjust the anesthetics concentration to keep the heart rate and blood pressure within 20% of the base line value throughout the anesthesia period.At the end of surgery, the first dose of paracetamol 1000 mg/100 ml intravenously (Medalgesic; ARABCOMED, Cairo, Egypt) was given to all patients before extubation. Then, reversal of neuromuscularblockade was performed with atropine (0.01 mg/kg) and neostigmine (0.05 mg/kg) given intravenously. After tracheal extubation, patients were transferred to the post-anesthetic care unit (PACU) where vital parameters were recorded every 1/2 h till complete recovery.

During the first 48 h, a standard analgesic regimen of paracetamol 1 g was given intravenously every 6 h to all patients. In addition, pain assessment in the ward was performed by nurses every 2 h and titrated doses of morphine (2 mg bolus at 10 min intervals) were given if patients reported pain (NRS was ≥3).

The postoperative data were collected by a senior resident (blinded to the study). The NRS pain scores was recorded at 30 min after the end of anesthesia (time = 0), all patients were able to answer questions and to rate their pain score at the end of 2, 4, 6, 12, 24 and 48 h postoperatively in the ward. Pain assessments were done at rest and with movement (after the patient completed a 90° logroll while in bed).

The time to first rescue analgesic, total morphine consumption at (24 h and 48 h) and the presence of side effects, such as headache, rash, nausea, vomiting, dizziness and drowsiness were recorded. The severity of postoperative nausea and vomiting (PONV) was graded on a four-point ordinal scale (I) not at all, (II) sometimes, (III) often or most of the time, and (IV) all of the time with vomiting [26]. Ondansetron, a rescue antiemetic, (4 mg) IV was given to all patients with PONV score more than II.

Patient satisfaction was measured at 24 h post-operatively using a numerical score of 1-4 (1 = poor, 2 = fair, 3 = good, 4 = very good).

After the study was completed, randomization and allocation were revealed for data analysis.Sample size estimation was made based on morphine consumption in a retrospective sample of 50 patients who was undergoing spinal surgery in our department. The sample size was calculated using power analysis (α = 0.05, β = 0.8) to detect 50% difference in morphine consumption between groups at 48 h post-surgery and was found to require at least 24 patients per group. Thus, we decided to include 30 patients per group to allow for possible drop-out.

### Statistical analysis

Data were presented as median (with inter-quartile range) or mean ± standard deviation. While qualitative data were presented as number (frequency distribution). Data such as ASA grade, sex distribution, Patient’s satisfaction, and Side effects were inferred by Chi-square test and fisher’s exact test. Data such as age, weight, height, mean duration of surgery, time of first rescue analgesic and total morphine requirement were inferred by ANOVA and post hoc Bonferroni test was used for in intergroup comparison. Differences in NRS scores were analyzed using the kruksal-wallis-test and the Mann–Whitney U-test was used for subsequent pairwise comparisons. The *p*-value of less than 0.05 was considered significance.

## Results

**Fig. 1:**
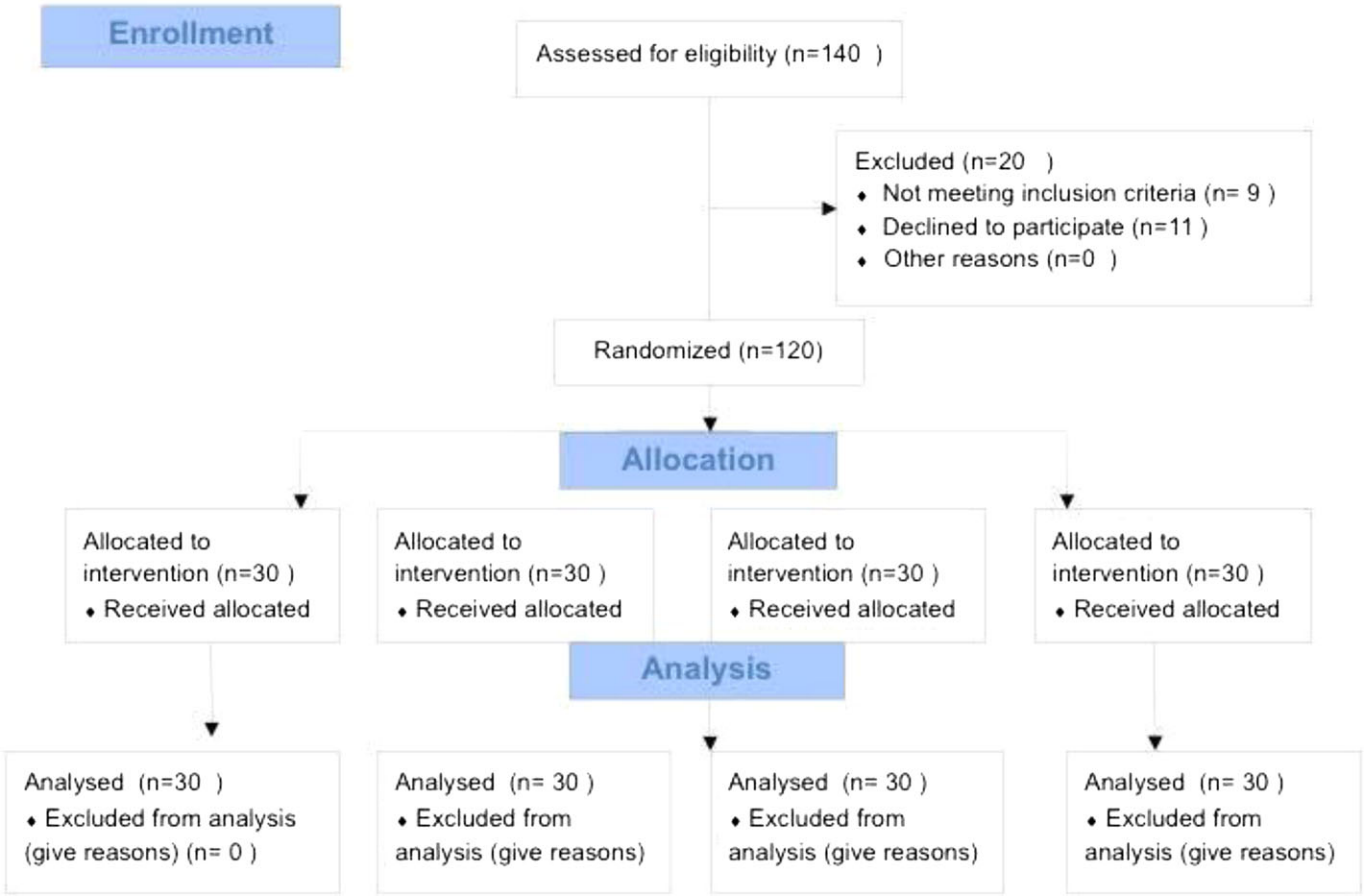
Flow diagram for participant

From November 1, 2015 to March 1, 2017, 131 consecutive patients who met the inclusion criteria were allocated for the study (Fig. 1). Eleven patients refused to participate. Therefore, 120 patients were randomized and included in the study. Characteristics of patients and surgical procedures for each group (Table 1) showed no significant differences between the groups.

**Table 1:** Characteristics of patients and surgical procedures in the four groups

Group Variable	Group (P) (*n* = 30)	Group (E) (*n* = 30)	Group (D) (*n* = 30)	Group (D/E) (*n* = 30)	*P* value
Age (years)	46.50 ± 8.74	45.26 ± 7.50	48.36 ± 9.80	47.50 ± 10.14	0.455
Male/Female (n)	15/15	17/13	18/12	16/14	0.471
Weight (kg)	81.23 ± 13.24	82.53 ± 12.90	80.60 ± 13.37	78.83 ± 16.78	0.794
Height (cm)	167.46 ± 8.50	165.53 ± 7.71	165.40 ± 8.21	165.00 ± 9.63	0.478
ASA (n)					
I	18	18	17	15	0.36
II	7	6	9	10	0.42
III	5	6	4	5	0.23
Duration of surgery (min)	109.9 ± 10.8	115.7 ± 9.8	113.2 ± 13.7	117.8 ± 9.7	0.65

Data are presented as Mean ± SD or number (n)

Placebo group (P), Etoricoxib group (E), Duloxetine group (D), Duloxetine/Etoricoxib group (D/E). Data were analyzed using ANOVA test with post hoc test (Bonferroni) and chi square test

### The morphine requirement

**Fig. 2:**
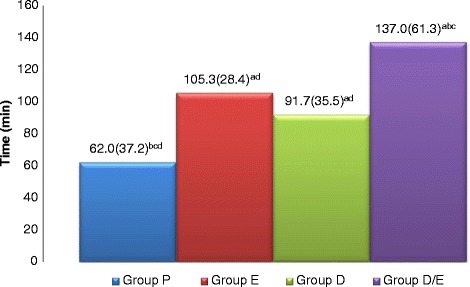
Time to morphine administration after surgery in the four groups as Mean(SD). Placebo group (P), etoricoxib (E), Duloxetine (D), Duloxetine/etoricoxib (D/E). a: when compared with P group. b: when compared with E group. c: when compared with D group. d: when compared with E/D group

The time to first rescue analgesic was significantly prolonged in (D/E)when compared with group D, group E and group P.There was a significant prolongation when groups E and D were compared with group P respectively with no significant difference between group E and group D (Fig. 2).

The morphine requirement at 24 h was statistically different between the four groups.

**Fig. 3:**
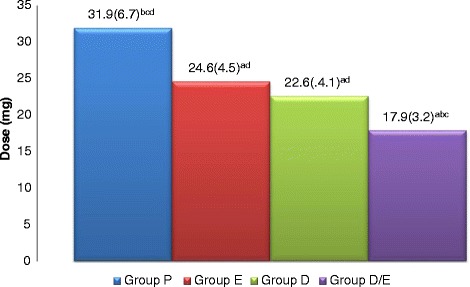
Morphine requirements at 24 h in the four groups as mean (SD). Placebo group (P), etoricoxib (E), Duloxetine (D), Duloxetine/etoricoxib (D/E). a: when compared with P group. b: when compared with E group. c: when compared with D group. d: when compared with E/D group

**Fig. 4:**
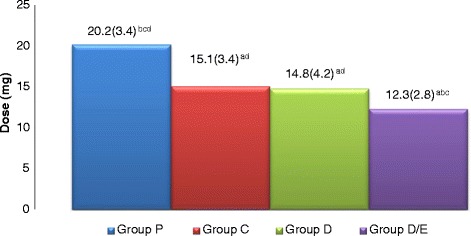
Morphine requirements at 48 h in the four groups as mean (SD). Placebo group (P), etoricoxib (E), Duloxetine (D), Duloxetine/etoricoxib (D/E). a: when compared with P group. b: when compared with E group. c: when compared with D group. d: when compared with E/D group

. There were significantly increased morphine requirements in the P group compared with E, D and D/E groups and significantly increased in E and D groups respectively when compared with D/E group with no significant difference between group E and group D (Fig. 3). At 48 h, total morphine requirement were still significantly increased in the P group compared with all groups with significant increases in both E and D groups when compared with D/E group with no significant difference between group E and group D (Fig. 4). But it was still significantly lower in the three groups at 48 h post-surgery when compared with those required at24 h.

### The pain score

With regard to pain scores at rest all time points, the duloxetine/etoricoxib (D/E) group had significantly lower pain scores when compared to placebo group P, while when it compared to etoricoxib group E, also when compared D/E with duloxetine group D (Table 2).

**Table 2:** Pain scores (NRS) at rest in the four groups

Group Variable	Group (P) (*n* = 30)	Group (E) (*n* = 30)	Group (D) (*n* = 30)	Group (D/E) (*n* = 30)	*P* value
At 0 h	5 (4-5.25)^bd^	4 (3-4) ^acd^	4 (3-5) ^bd^	3 (3-4) ^abc^	0.0001
At 2 h	4 (3-5)bd	3 (3-4) ^acd^	4 (3-5) ^bd^	3 (3-3)^abc^	0.0001
At 4 h	4 (3-5) ^bd^	3 (3-4) ^acd^	3 (3-4) ^bd^	2 (2-3)^abc^	0.0001
At 6 h	3 (3-4) ^bd^	3 (2-4) ^ad^	2.5 (2-3)^d^	2 (1-3) ^abc^	0.0001
At 12 h	3 (3-3) ^bd^	3 (2-3) ^ad^	3 (2-3)^d^	2.5 (1-3) ^abc^	0.0001
At 24 h	3 (2-3) ^bcd^	2(2-3) ^ad^	2.5 (2-3) ^ad^	2 (1-2)^abc^	0.0001
At 48 h	3 (2-3) ^bcd^	2 (2-3) ^ad^	2 (2-3) ^ad^	2 (0.75-2)^abc^	0.0001

Placebo group (P), Etoricoxib (E), Duloxetine (D), Duloxetine/Etoricoxib (D/E)

Data are presented as median (interquartile range). Data were analyzed by Mann–Whitney U-test and kruksal-wallis test and *P* < 0.05 is considered significant

a: when compared with P group

b: when compared with E group

c: when compared with D group

d: when compared with D/E group

The pain score in group E was significantly decreased at most time periods when compared to group P at 0, 2 and 4 h at rest when compared with group D. The pain score in group D was significantly decrease at 24 and 48 h compared to group P (Table 2).

While on movement pain was significantly decreased in D/E at all times when compared togroup P and when it compared to group E and when it compared to group D with no significant difference between other groups on movement (Table 3).

**Table 3:** Pain scores (NRS) on movement in the four groups

Group Variable	Group (P) (*n* = 30)	Group (E) (*n* = 30)	Group (D) (*n* = 30)	Group (D/E) (*n* = 30)	*P* value
0 h	5 (5-6.25) ^d^	5 (5-6) ^d^	5 (5-6) ^d^	5 (4.5.25) ^abc^	0.013
After 2 h	5 (5-6) ^d^	5 (4-6) ^d^	5 (5-6) ^d^	5 (4-5) ^abc^	0.002
After 4 h	4 (4-5) ^d^	4 (4-5) ^d^	4 (4-5) ^d^	4 (3-4) ^abc^	0.019
After 6 h	4 (3-5) ^d^	4 (3-5) ^d^	4 (4-5) ^d^	4 (3-4) ^abc^	0.007
After 12 h	4 (3-5) ^d^	4 (3-4) ^d^	4 (3-4.25) ^d^	3 (3-4) ^abc^	0.030
After 24 h	4 (3-5) ^d^	4 (3-4.25) ^d^	4 (3-4) ^d^	3 (2.75-4) ^abc^	0.059
After 48 h	3.5 (3-4) ^d^	3 (3-4) ^d^	3.5 (3-4) ^d^	3 (2.75-4) ^abc^	0.049

Placebo group (P), Etoricoxib (E), Duloxetine (D), Duloxetine/Etoricoxib (D/E)

Data are presented as median (interquartile range). Data were analyzed by Mann–Whitney U-test and kruksal-wallis test and *P* < 0.05 is considered significant. a: when compared with P group

b: when compared with E group

c: when compared with D group

d: when compared with D/E group

### Patients’ satisfaction

The percentage of patients’ satisfaction (excellent) shows significant differences between the four groups at 24 h (Table 4) with no significant differences between the three groups at 48 h.

**Table 4:** Patient’s satisfaction in the four groups at 24 h

Group Patient satisfaction	Group(P) (*n* = 30)	Group(E) (*n* = 30)	Group(D) (*n* = 30)	Group(D/E) (*n* = 30)	p
ExEellent	9 (30%)	12(40%)	11(36.7%)	21 (63.3%)*	0.004
Good	9(30%)	10(33.3%)	9(30%)	5(23.3%)	0.237
Fair	8(26.7%)	5 (16.5%)	6(20%)	2(6.7%)	0.069
Poor	4(13.3%)	3(10%)	3(10%)	2(6.7%)	0.933

Data are presented as number (%). Data were analyzed using chi square. Placebo group (P), Etoricoxib (E), Duloxetine (D), Duloxetine/Etoricoxib (D/E). *P* < 0.05 is considered significant

*P = 0.016 when compare with P

The most common adverse effect expected by patients in the study was nausea and vomiting grades III and IV. There was a significant increase in percentage of patients in group P (43.3%) when compared with group D/E (16.6%) and who reported nausea and vomiting. All complained patients responded to i.v.ondansetron. No statistically significant differences were noted between groups with regard to adverse effects (Table 5).

**Table 5:** Side effects in the four groups

Group Side effect	Group(P) (*n* = 30)	Group(E) (*n* = 30)	Group(D) (*n* = 30)	Group(D/E) (*n* = 30)	p
PONV (%) IIII&IV	13 (43.3%)*	7 (23.3%)	7 (23.3%)	5 (16.6%)	0.027
Somnolence	1 (3.3%)	1 (3.3%)	2 (6.7%)	3 (10%)	0.225
Pruritus	5 (16.7%)	4 (13.3%)	3 (10%)	3 (10%)	0.390
Dizziness	1 (3.3%)	2 (6.7%)	4 (13.3%)	3 (10%)	0.239
Headache	6 (20%)	3 (10%)	5 (16.7%)	4 (13.3%)	0.907

Data are presented as number (%). Data were analyzed using chi square test and fisher’s exact test. Placebo group (P), Etoricoxib (E), Duloxetine (D), Duloxetine/Etoricoxib (D/E). *P* < 0.05 is considered significant. *P = 0.024 when compare P and D/E

## Discussion

To our knowledge, there have been no studies evaluating the combination of selective COX-2 inhibitors (etoricoxib) and a selective serotonin and norepinephrine reuptake inhibitor (SNRI) (duloxetine) after spine surgery. Therefore, we decided in this study to use this regimen based on the results of previous clinical trials. A number of reports have demonstrated success with either the use of etoricoxib [18–20, 23] or duloxetine [8–11] with less reported success about the efficacy of their combination in humans. Sun et al., [24] reported that pretreatment with an intraperitoneal injection of duloxetine and celecoxib produced synergistic analgesia and could attenuate pain in mice 1 h after formalin injection.

Duloxetine is a selective SNRI that is prescribed for treatment of depression and anxiety disorders [27]. It is also efficacious in treating pain in diabetic neuropathy and fibromyalgia [6]. The mechanism of its analgesic action could be explained by a combined central and peripheral pain modulating role [28] through the effect of serotonin and norepinephrine on descending inhibitory pain pathways in the brain and spinal cord [29] and activation of some cerebral prefrontal areas [5]. Also it has a antinociceptive effect through Na + channel blocks [30] with antihyperalgesic effects through the inhibition of the neuronal cell firing resulting from peripheral injury [31]. Therefore,duloxetine has a great role in management of neuropathic pain and reducing postoperative pain. In addition, it may improve the depression and anxiety that are common during the perioperative period [32].

In this randomized study, despite the fact that each of the two drugs separately could not produce analgesia during movement, their combination induced significant reduction in pain score at rest and on movement over the study time points and also improved patients satisfaction at 24 h postoperatively. Although, each of the drugs separately were able to prolong the duration of first rescue to analgesia and reduce postoperative morphine consumption, the combination also remained significantly effective when compared with them. Therefore, this may accelerate the rehabilitation and reduce postoperative morbidity [33].

The analgesic effect of antidepressants is typically seen after 7 to 14 days, therefore It’s commonly used for chronic pain [34]. However, some investigators use duloxetine immediately preoperatively for acute pain management [8, 10]. In our study, we demonstrated that two doses of (60 mg) duloxetine 1 h before surgery and after 24 h could reduce opioid consumption with no significant effect on early postoperative pain score. Our result was comparable to Ho et al. [8] who assessed the use of two doses of duloxetine on pain scores postoperatively following knee arthroplasty. Also, Castro Alves et al. [10] examined the same regimen in patients undergoing abdominal hysterectomies and recently, Bedin et al. [11] performed the same assessment after spine surgery. On our study, the first dose of duloxetine was given 1 h before surgery. In contrast, Nasr [9] gave the first dose of duloxetine 60 mg 2 days before surgery in patients undergoing mastectomy and recorded lower pain scores in the duloxetine group compared to a control group at the study period.

Although etoricoxib has been shown to have significant analgesic efficacy during pain at rest when compared to thecontrol group in our results, there was no effect on pain score on movement. These results resembled those of Rawal et al. [35] where they evaluated the effect of etoricoxib (90 or 120 mg), versus ibuprofen (1800 mg) on postoperative pain following knee replacement and concluded that etoricoxib(90 and 120 mg) was significantly effective in reducing pain at rest and also reduced morphine consumption when compared to placebo with no significant effect on movement. Also Lierz et al. [23] used 120 mg of etoricoxib or placebo 1 h before induction of general anesthesia in knee arthroscopy surgery. They recorded similar results, showing reduction in pain only at rest and reduction in morphine consumption.

Opioids are considered the drug of choice for management of postoperative pain but it is difficult to induce an optimum analgesia without significant side effects [36]. Therefore, we suggest in our study that short-term duloxetine treatment in combination with etoricoxib may be a good adjuvant for decreasing the need for opioids in order to alleviate postoperative pain without significant adverse effects. In our results there were 13 patients complaining of nausea and vomiting in the placebo group with significant difference when compared to D/E group. There were no incidences of other adverse effects, such as sedation, dizziness, somnolence, pursuits or headache.

In this study we evaluate the acute postoperative pain not the chronic pain examined in previous studies [8, 9, 37] because our study was on a group of patients complaining from chronic back ache with high incidence of postoperative failed back pain syndromewith multifactorial conditions which may affect up to 10 to 40% of patients [38].

## Conclusion

The present study demonstrates that the perioperative administration of the duloxetine/etoricoxib combination reduces postoperative pain, beside the need for morphine at 24 and 48 h after lumbar spine surgery, and the opioid-related side effects more effectively than either drug alone. Duloxetine/etoricoxib combination may thus be a useful adjuvant to be used along with opioid as part of a multimodal analgesia in the acute postsurgical setting.

Concerning limitations to our study, there are some to be applied. First it is not possible to prove that the combination of duloxetine and etoricoxib has more than just an additive effect because we did not make a full dose-response study nor associated ED50s. The second limitation of our study is that we evaluated a possible effect of duloxetine on acute postsurgical pain alone and not on the chronic one.

## Abbreviation

COX: Cyclooxygenase
ECG: Electrocardiogram
HR: Heart rate
MABP: Mean arterial blood pressure
NRS: Numeric rating scale
NSAIDs: Non-steroidal anti-inflammatory drugs
SPO2: oxygen saturation
SSNRI: Selective serotonin and norepinephrine reuptake inhibitor
